# Sulphuric Acid in Cholera

**Published:** 1853

**Authors:** 


### *Sulphuric Acid in Cholera.*

In the summer of 1851 (writes Dr. Fuller), the attention of the profession was directed to a strange and newly-discovered property of sulphuric acid. That which had been regarded as a frequent source of belly-ache and purging was vaunted as a certain cure for these evils, and medical men were called upon to examine and report upon this novel anti-cholera specific. It was not, however, until the month of September, 1851, that I had an opportunity of testing its efficacy in the cure of true choleraic symptoms. In that month, two most urgent cases of choleraic diarrhœa, attended by extreme collapse, violent and incessant cramp, pinched features, rice-stool evacuations, and matters resembling chicken-broth rejected from the stomach, were admitted about the same time into St. George's Hospital. Had theso cases occurred in the autumn of '49, they would have been designated cases of Asiatic cholera; they differed in no respect from genuine cholera, and were entitled cases of choleraic diarrhœa, simply because cholera was not then prevalent as an epidemic. In both these instances I administered sulphuric acid with complete success: and, as the action of the remedy could be attested by pupils and others, who witnessed the success of the treatment, I published in the *Medical Times and Gazette* (January 10th, 1852), a detailed account of the effects observed, together with the general result of my experience. It amounted to this—that the dilute sulphuric acid of the pharmacopœia, administered in half-drachm doses, repeated at short intervals, has a powerful and immediate influence over the disease. The vomiting and purging cease, and the cramps subside, sometimes after the second, more usually after the third or fourth, doses of the medicine. The attack may leave the patient weak, and usually does so, in proportion to its severity, and the length of time which has elapsed before the administration of the remedy is commenced; but within three or four hours, the disease is arrested, and convalescence follows speedily. Indeed, in ordinary cases, the change is effected with such marvellous rapidity, that he who in the morning is so utterly prostrate, in the evening experiences little ill effect from his fearful seizure.

I had hoped that this circumstantial report, backed as it was by the result of my own experience, extending at that time to twenty-seven cases, and as it has since been by the concurrent testimony of several practitioners, in different parts of the country, would have led to a recognition of the extreme value of this treatment, in all instances of choleratic diarrhœa, if not of cholera; at all events, it seemed but fair to presume that testimony so decided, in favor of a remedy so cheap, so harmless, so extremely simple, so uniformly successful, and producing its successful and striking results, within so short a time of its administration, would at least have insured a trial of its virtues.

My own conviction is, that in sulphuric acid we have an antidote—a specific—against choleratic diarrhœa, if not against the worst forms of cholera, as powerful, as energetic, and as certain in its effect, as in cinchona bark, or quinia, against a paroxysm of ague. In bilious diarrhœa, and in certain chronic diarrhœas, it is of little or no avail; and, in some, though in very few, according to my experience, it gives rise to pinching pain in the abdomen; but in epidemic diarrhœa, in acute autumnal diarrhœa, if I may so term it, and in more decided choleraic diarrhœa, I have known no single instance of its failure; indeed, the more choleraic the diarrhœa, the more speedily are its curative effects produced. This fact will appear of greater value when it is known that I have notes of its administration in above ninety such cases, many of which have occurred among my own patients at the hospital or elsewhere, and some in the practice of my friends.

With this, however, even more than with other remedies, success depends upon the mode of administration. Given at intervals of three or four hours, it fails in exerting any curative influence in severe cases, and is seldom of much avail, even in milder cases; and to insure its beneficial effects, it should be exhibited in full doses, repeated at much shorter intervals. When I first began to give it, I ordered two doses to be taken within the first hour, and a dose to be repeated every hour afterwards. But recent and more extended experience, together with the close and eager observation of its influence on my own person, has convinced me that, even in ordinary cases, twenty minutes form a sufficient interval between each dose. In severe cases, five or six doses may be given with advantage within an hour; and should any dose be rejected by the stomach, another should be administered at once.* Rarely, however

---

* Mr. Buxton, of Great George Street, Westminster, to whom the profession is greatly indebted for having been the first to call attention to the anti-choleraic properties of sulphuric acid, recommends the administration of half drachm doses every quarter of an hour in severe cases. If those gentlemen who make trial of the remedy so strongly recommended by Mr. Buxton would use it as they ought, in the manner, and in the dose he directs, and administer it only only in proper cases, we would seldom, if ever, hear of its failing to arrest the course of the disease. In every instance is any dose rejected except the first, and the rejection of that is by no means common. While loathing the sight of other liquids, the patient, after he has tasted the first dose of the medicine, very generally craves for its repetition, and drinks it with avidity.

As the stomach is very irritable, and the acid taken in simple water is, under circumstances, extremely palatable, I never mix it with syrup or other matters; and I strongly suspect the warm aromatic spices, with which some of my friends have flavored it, are additions by no means agreeable to the patient. Unfortunately, I have had some personal experience in this matter, and so strongly was my distaste to the medicine excited by such flavorings, that for the future, whenever I am the patient, I shall certainly put my veto upon them.

The effects produced by this remedy are very remarkable. Sometimes, after the second dose, more commonly after the third, and almost always after the fourth dose of the medicine, the patient experiences a grateful sense of warmth at the epigastrium; heat returns to the extremities, the nausea and vomiting immediately cease, even if they have not been arrested already; the purging is stayed, the cramps subside, and the countenance resumes its natural appearance. Very generally perspiration breaks out, and the patient goes to sleep, and awakes refreshed, though feeling somewhat weak. The other symptoms betoken a like amendment. The tongue, which before was dried and furred, or shrivelled, becomes moist and slightly coated: the evacuations from the bowels assume a healthy color, and are accompanied by an unusual discharge of bile; and the pulse regains its normal steadiness.

Such being the case, it is unnecessary, in mild or ordin-

---

within my own knowledge in which it has failed in its efficacy, the blame has been chargeable upon those who have administered it, rather than upon any want of virtue in the remedy itself. They have either given it in insufficient doses, or else in cases of bilious diarrhœa, with foul breath and loaded bowels, which would have been benefitted by a brisk purge of calomel and rhubarb.

ary instances, to have recourse to any further treatment. With due precaution as to over-exercise and fatigue, and strict attention to the amount and nature of the diet during the next few days, so as to allow the stomach to recover its wonted energy before its powers are again fully taxed, the patient usually recovers without let or hindrance. The bowels act regularly without the assistance of medicine, and the ejections are of a healthy color and consistence. But in severe cases, if care be not taken to maintain the good effects already produced, a relapse may occur, or recovery may prove tardy. The patient, though no longer troubled with diarrhœa, may digest imperfectly, and suffer greatly from nausea. For some time after these severe attacks, the secretion of bile takes place irregularly, and the bowels become costive or irritable, and the motions dark-colored and offensive. Hence it is sometimes expedient to follow the sulphuric acid by two or three grains of blue pill, or a grain of calomel, administered at bed-time for a few successive nights, taking care during the day to impart tone to the stomach by the exhibition of some light, bitter infusion, combined with potash and ammonia, small doses of tincture of rhubarb, and, if necessarry, a little chalk. By some such means as these, all unpleasant after symptoms are prevented, and the patient proceeds steadily to convalescence.

On several occasions, it has occurred to me to try whether the same effects might not be produced by continuing the administration of sulphuric acid at intervals of six or eight hours. But the result of the experiment has not been encouraging. In some few instances the patient has improved steadily, but occasionally he has experienced a relapse, and has generally been longer arriving at perfect health than under the plan already indicated. Hence, as soon as the urgent symptoms have been subdued—*i. e.*, after the first few hours—I usually discontinue the acid, and, if necessary, have recourse to the treatment above recommended as most conducive to the maintenance of its good effects.

